# Contribution of Interfacial Bonding towards Geopolymers Properties in Geopolymers Reinforced Fibers: A Review

**DOI:** 10.3390/ma15041496

**Published:** 2022-02-17

**Authors:** Muhd Hafizuddin Yazid, Meor Ahmad Faris, Mohd Mustafa Al Bakri Abdullah, Marcin Nabiałek, Shayfull Zamree Abd Rahim, Mohd Arif Anuar Mohd Salleh, Marwan Kheimi, Andrei Victor Sandu, Adam Rylski, Bartłomiej Jeż

**Affiliations:** 1 Geopolymer & Green Technology, Centre of Excellence (CEGeoGTech), Universiti Malaysia Perlis (UniMAP), Kangar 01000, Malaysia; meorfaris@unimap.edu.my (M.A.F.); shayfull@unimap.edu.my (S.Z.A.R.); arifanuar@unimap.edu.my (M.A.A.M.S.); 2 Faculty of Chemical Engineering Technology, Universiti Malaysia Perlis (UniMAP), Kangar 01000, Malaysia; 3 Faculty of Mechanical Engineering Technology, Universiti Malaysia Perlis (UniMAP), Kangar 01000, Malaysia; 4 Department of Physics, Częstochowa University of Technology, 42-200 Częstochowa, Poland; nmarcell@wp.pl (M.N.); bartek199.91@o2.pl (B.J.); 5 Department of Civil Engineering, Faculty of Engineering—Rabigh Branch, King Abdulaziz University, Jeddah 21589, Saudi Arabia; mmkheimi@kau.edu.sa; 6 Faculty of Materials Science and Engineering, Gheorghe Asachi Technical University of Iasi, 71 D. Man-geronBlv., 700050 Iasi, Romania; sav@tuiasi.ro; 7 Institute of Materials Science and Engineering, Faculty of Mechanical Engineering, Lodz University of Technology, Stefanowskiego 1/15, 90-924 Lodz, Poland; adam.rylski@p.lodz.pl

**Keywords:** geopolymers concrete, fiber interfacial, compressive strength, fly ash

## Abstract

There is a burgeoning interest in the development of geopolymers as sustainable construction materials and incombustible inorganic polymers. However, geopolymers show quasi-brittle behavior. To overcome this weakness, hundreds of researchers have focused on the development, characterization, and implementation of geopolymer-reinforced fibers for a wide range of applications for light geopolymers concrete. This paper discusses the rapidly developing geopolymer-reinforced fibers, focusing on material and geometrical properties, numerical simulation, and the effect of fibers on the geopolymers. In the section on the effect of fibers on the geopolymers, a comparison between single and hybrid fibers will show the compressive strength and toughness of each type of fiber. It is proposed that interfacial bonding between matrix and fibers is important to obtain better results, and interfacial bonding between matrix and fiber depends on the type of material surface contact area, such as being hydrophobic or hydrophilic, as well as the softness or roughness of the surface.

## 1. Introduction

The global construction industry is increasingly using Ordinary Portland Cement (OPC), resulting in a significant increase in greenhouse gas emissions, making it necessary for researchers to take serious preventive measures. As ecologically acceptable alternative adhesives are required, and because a huge number of waste materials and by-products are disposed to landfills, the development of non-cementing materials has obtained significant research and application such as sustainable technology [1,2].

Furthermore, cement is widely used in the building industry all over the world, and rising investment in infrastructure for developing countries would increase cement use. It is well known that climate change is a big problem for the environment and the release of carbon dioxide (${\mathrm{CO}}_{2})$, methane, and other gases will cause the greenhouse effect [3]. Cement manufacture alone emits 13.5 billion tons of ${\mathrm{CO}}_{2}$ in the construction industry, and loads of greenhouse gas emissions each year, accounting for 7% of global carbon dioxide emissions, which is a significant amount [4]. In OPC, cement combines all fundamental fabrics extremely well. With an emanation rate of roughly 700–900 kg per ton, cement has the highest carbon emissions. Several studies have attempted to reduce cement content in concrete blends by partially or completely replacing cement with mineral admixtures or mechanical by-products, with the goal of reducing concrete ${\mathrm{CO}}_{2}$ outflows [5,6,7].

Reinforced concrete (RC) structures have been widely constructed all around the world. Nonetheless, traditional RC systems composed of Portland cement and steel have the disadvantages of marine infrastructure, including low lifespan due to hydration degradation. Products and steel bars are corroded and are incompatible with sea sand and sea water, resulting in short life and unsustainable operation [8].

Geopolymers are considered a green product because the carbon dioxide released is less than 80% of the conventional cement, such as lime and Portland cement [9,10]. Fly ash (FA), Slag (SG), and Meta Kaolin (MK) are only a few examples of geopolymer-based materials comprising of silica (${\mathrm{SiO}}_{2}$) and alumina (${\mathrm{Al}}_{2}O_{3}$) as the major components that react with a concentrated alkaline solution to generate heat energy, consequently speeding up the processes [5,11]. Geopolymers made from waste resources, such as fly ash, can be utilized as a raw material for geopolymers concrete to cut costs [12,13].

Fly ash, blast furnace slag, copper, and zinc slag are examples of industrial waste products that can be used as aluminosilicate source geopolymers synthesis since$\;{\mathrm{SiO}}_{2}$ and ${\mathrm{Al}}_{2}O_{3}$ are the major oxides in the process. Due to its widespread availability and contribution to the production of high-quality binders, fly ash is regarded as one of the most pozzolanic by-product materials in the building sector. As a result, several geopolymer researchers have looked at its mechanical properties, durability, and microstructural composition [14,15,16,17,18].

Geopolymers have mechanical and physical qualities that are quite comparable to ordinary OPC. Geopolymers that have high compressive strength but low tensile strength have brittle characteristics [19,20]. Fiber-reinforced concrete is a technique for improving the brittleness of concrete by combining particular fibers with various materials to obtain the desired qualities [21].

Due to their properties, ductility materials have a high energy absorbency, which means they have a low modulus of elasticity (MOE), which varies depending on the material. The area under the graph shows how much energy is absorbed, depending on the material’s resistance value; however, when it comes to the reinforced fibers, the resistance value of the material will decrease due to other materials that affect the properties of energy transfer during fracture or cracking [22].

Steel fibers (SF), carbon fibers (CF), polymer fibers (PF), and natural fibers (NF) have both high and low modulus (metallic) (non-metallic) depending on the kind of material and geometry [23]. Because of the high brittleness of geopolymers, introducing fibers improves fractural strength and helps overcome fracture toughness. Fibers can control and prevent cracking by performing tasks such as debonding, sliding, and pull-out [24].

The characteristics of fibers that influence interface bonding and the ability to load transfer from matrix to fibers determine the bonding strength between matrix-fiber. The performance of geopolymers reinforcements is determined by fibers characteristics, fibers content, curing time, geopolymers technique, and the type of raw material used to make geopolymers [25,26]. According to an earlier study, geopolymers reinforced Polypropylene (PP) fibers can produce light geopolymers concrete due to the low density of PP fibers compared to geopolymers [27,28].

Many applications, such as the alignment of fibers, require a thorough understanding of the interactions between droplets and fibers. The apparent contact angle (ACA) of a droplet with fibers might differ greatly from the Young–Laplace contact angle (YLCA) of a small droplet of the same liquid placed on a flat surface composed of the same material, according to numerous pioneering studies of droplet–fibers interactions [29,30]. Two distinct conformations have been seen depending on fiber diameter, surface energy, droplet volume, and droplet surface tension. If a droplet has been deposited on a fiber, the term “droplet” is used. The initial conformation is that of the barrel. Larger droplets (compared to fiber) or larger droplets (relative to fiber) are more likely to have this shape when the YLCA is not too high.

Because they are less expensive than steel fibers, polymer-based fibers are being utilized to strengthen various concrete kinds. Polyolefin is a polymer-based fiber that improves flexural toughness, fatigue strength, and impact resistance in concrete composites while also preventing crack propagation [31]. The use of two-part and multipart hybrid fibers in concrete composites to improve various qualities has sparked a lot of interest in recent years. Fibers of various lengths made from the same material are joined in these hybrid fibers. FRGPC (fiber reinforced geopolymers concrete) is a new type of geopolymers concrete (GPC) that has been the subject of several recent studies to identify its potential benefits and downsides.

Many studies have been conducted on geopolymer-concrete reinforced fibers, such as polymers and steel fibers, which have qualities that are superior to polymer fibers, but polymer fibers are still in demand due to their low cost and light weight. Many research hybrid or reinforced fibers have recently gained popularity. These hybrid fibers use different types of form or length to provide significant flexural strength improvements over GPC. The effect of hybrid PP and steel FRGPC on compressive and flexural strength is about 30% and 200% higher than the GPC, respectively [32]. According to prior studies on geopolymer concrete reinforced fibers with 6- and 12-mm long steel fibers, the shorter fiber is better at controlling tiny cracks, whereas the longer fibers provide ductility in large cracking situations. A hybrid fiber arrangement was shown to provide the best fracture control properties. Asrani et al. investigated slag-based FRGPC with PP (13 mm long), glass (15 mm long), and 3D-steel (60 mm long) fibers with 0.3, 0.3, and 1.6 percent volume content, respectively, in single and hybrid fiber GPC configurations, as well as single and hybrid fiber GPC configurations [33].

Ganesan investigated the durability characteristics of plain and fiber-reinforced geopolymers concrete in comparison to Portland cement concrete. It came to the conclusion that plain and fiber-reinforced geopolymers concrete produced better outcomes than conventional concrete in general, and that the addition of fibers resulted in a better improvement in terms of durability attributes [34]. The correlation interface bonding of fibers and matrix to fiber properties such as dimension, fiber type, and materials will be discussed in this review paper. The properties of fibers will be discussed in the following session.

## 2. Fibers

To boost the flexural strength and energy absorption of geopolymers composites, fibers in various forms have been employed as reinforcements. In general, when choosing fiber for reinforcement in cementitious and geopolymers composites, three main criteria must be considered: material qualities that are compatible with the application, such as lightweight or high impact, adequate fiber–matrix interaction to convey stresses, and an optimal aspect ratio to ensure good post-cracking behavior. Before examining the composite activity of fiber and geopolymers, let us have a look at the material and geometric qualities of the fibers that will be used [35].

### 2.1. Fiber Types and Properties

A fiber’s material qualities are often more important than binders in determining the performance of fiber-reinforced geopolymer composite. The polypropylene fiber, for example, has a weak fiber/binder interaction regardless of binder type, geopolymers, or cement, lowering the composite’s compressive strength [36,37,38]. Carbon-based, steel, inorganic, natural, and polymers fibers are divided into five primary classes in this study to discuss the significance of content attributes.

Steel fibers are used in cementitious composites because of their great mechanical strength, flexibility, and availability. ASTM A820-16 standard specifies five types of steel fibers for specific applications: smooth or deformed cold-drawn wire, smooth or deformed cut sheet, melt such as dynamic loading extracted, mill cut, and modified cold-drawn wire steel fibers, all of which are small enough to be randomly dispersed in concrete. Steel fibers can have tensile strengths and ultimate elongations ranging from 310 to 2850 MPa and 0.5 to 3.5 percent, depending on the type of material and the manufacturing process [39,40,41]. Any one of ten specimens’ minimum tensile strength must be greater than 310 MPa, and the average tensile strength must be greater than 345 MPa, according to ASTM A820-16. Metallic fibers have a corrugated surface due to their malleability and production procedures, resulting in significant fiber–binder contact [42]. Steel fibers, a commonly used construction material, have a number of advantages, but the most significant disadvantage is corrosion [43,44]. To overcome this issue, stainless steel alloys such as austenitic, ferritic, martensitic, duplex, and precipitation harden able steels, as well as sacrificial coating composites such as copper/zinc-coated steels, are used to resist corrosion on the steel fibers material [45].

Polymers are made up of many chains of tiny monomer units that are bounded together by intermolecular interactions [46]. Polymer characteristics are influenced by their intermolecular interactions. Polymers are categorized as crystalline (more than 80% crystallinity), semi-crystalline (greater than 80% crystallinity), or amorphous (less than 80% crystallinity) (less than 10 percent crystallinity) [34,47]. The mechanical characteristics, stiffness, environmental resilience, and surface roughness of polymers can all be improved by increasing crystallinity. Polymeric fibers can also be classified as synthetic or natural based on their source materials and manufacturing process.

Geometrical metrics such as fiber cross-section and length, area of a fiber’s surface in a composite unit volume, and cross-sectional area fibers over a particular plane of the fiber-reinforced matrix are all important factors to consider when evaluating fiber efficiency, in addition to material properties.

Fibers, whiskers, and particles are the three types of reinforcement [48]. As the diameter of a fiber increases, its mechanical strength and modulus decrease. Glass fibers, polyvinyl alcohol (PVA), wires, inorganic, alumina fibers and whiskers fibers [48], and polycaprolactone [49] have all demonstrated this. This can be explained by the fact that big diameter fibers have a higher likelihood of defects and flaws compared to single-crystal whiskers or finer fibers [50]. Surprisingly, this effect is more pronounced in stronger materials.

Individual fibers can take on a nearly infinite number of geometric shapes. Where manufacturing methods allow, it is recommended to pre-deform the fibers to contribute mechanical anchoring to the fiber–binder interaction [51]. Hooks, paddles, and buttons can be used to attach the deformed part to the end of the fibers, or longitudinal deformation can be achieved by indenting, crimping, and twisting the fibers. Similarly, the cross-section of fibers can be prismatic, rounded, or polygonal, with a surface that is smooth or corrugated and uneven. During the mixing process, multifilament and monofilament networks (or bundles) separate, as well as a varying cross-section along the length of the fibers. Furthermore, the cross-section structure can be solid, coated (such as copper-coated steel fibers), or a combination of the two [49], with shielded fibers (e.g., SiC fibers and SiC coated carbon fibers [52]), and tubular structures (e.g., flax fibers and hemp fibers) [53]. The numerical simulations will be discussed in the next section.

### 2.2. Numerical Simulations

**Figure 1:**
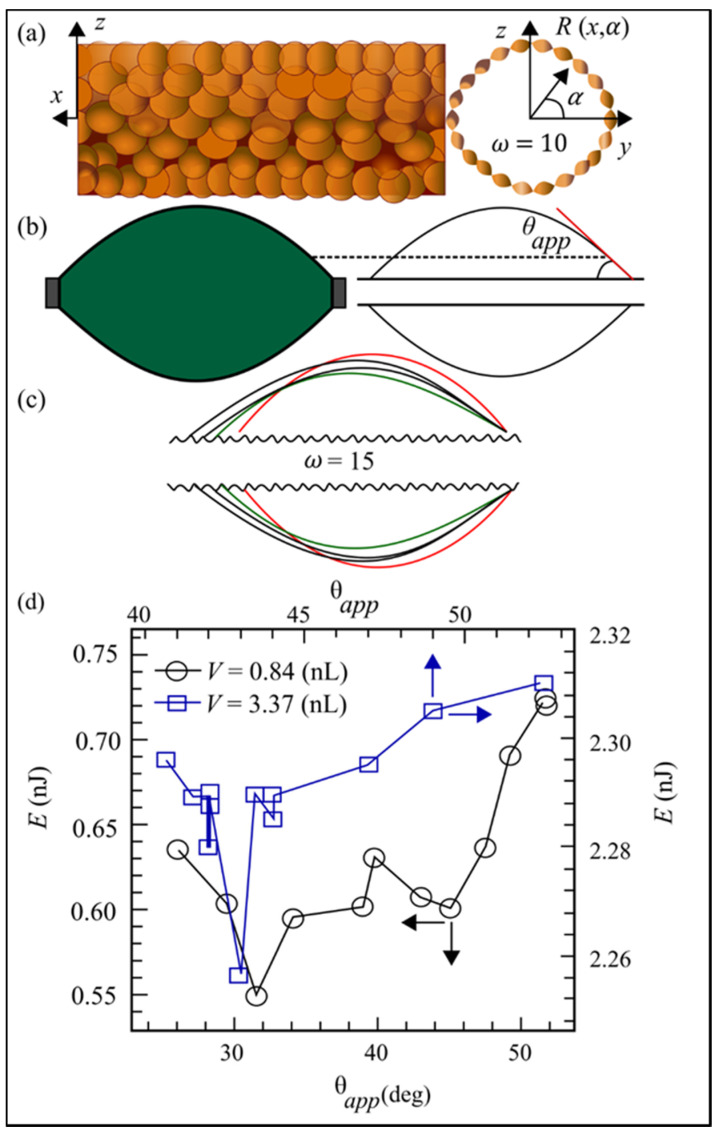
Our simulated crude fibers are displayed through side and cross-sectional views (**a**). The point of inflection and apparent contact angle are depicted in (**b**). In (**c**) the volume *V* = 0.84 nL on rough fibers with *rf* = 15 µm, $\theta_{YL}$ = 30 and x = 15. In (**d**), the surface energy of droplets is plotted against apparent contact angle for droplet volumes of *V* = 0.84 nL (black symbols) and *V* = 3.37 nL (blue symbols) [59].

The surface energy minimization approach of the surface evolver (SE) finite element model is used to simulate the 3-D geometry of droplets on rough fibers. SE has been demonstrated to be accurate in forecasting the stability of the air–water interface. In this section, the equations for creating fibers with any 3-D roughness are presented, and then an expression for the energy of droplets placed on such fibers is developed. To the best of our knowledge, there has been no research on using mathematical functions to simulate or quantify fiber roughness. Although the shape and arrangement of the actual roughness are arbitrary, for the sake of simplicity, the sine roughness (the sine function is also used to describe the roughness on the plane) is used [54,55,56,57]. In each cross-section of the fibers, the rose function (sine curve in polar coordinates) can cause sinusoidal roughness [58]. By multiplying this equation by another sine function along the fiber axis, the 3-D roughness of the fibers can be yielded, as shown in Figure 1a.

Consider the case where the fibers have sinusoidal roughness in the axial and preferred directions in the x-direction, as shown in Figure 1a.

(1) $$R\left(x,\alpha \right)-r_{f}\;[1+asin(\frac{2\pi }{\lambda r_{f}})\sin\left(\omega \alpha \right)]=0$$

where $r_{f}$ is the smooth fiber radius, R(x,α)= $\sqrt{y^{2}+z^{2}}$ is the rough fibers’ radius inside the locality at any given site, d $\alpha =Arctan_{y}^{z\;}$ is the angle of the position. The roughness amplitude equation is, λ is wavelength roughness, and $\omega =\frac{2\pi }{\lambda }$ of roughness peak’s angular frequency (see Figure 1a. Dimensionless roughness amplitude is a term that is used for convenience as $=\frac{\alpha }{r_{f}}$ (note that b = a if $r_{f}$ = 1). By limiting the total energy of the droplet–fibers system, SE is utilized to achieve a balanced 3-D form of droplets deposited on coarse fibers. The total free energy E for a single droplet–single fiber can be stated as

(2) $$E=\sigma_{LG}A_{LG}-\sigma_{LG}\int_{A_{SL}}\cos\theta_{YL}dA+\int phgdV\;$$

where $\sigma_{LG}$ determines the liquid’s surface tension, and $A_{LG}$ and $A_{SL}$ are the liquid–gas and solid–liquid regions, respectively. Among these, h denotes the vector change in the position of the droplet center of mass to physical force (zero if no external force is present), and g is the physical force per unit mass, ρ representing the liquid density, dA and dV are the components that represent the area and volume, respectively. The influence of fibers on geopolymers is presented in the next section.

## 3. Effect of Fibers on the Geopolymers Composite

This section will discuss the impact of fiber diameter and geometry on the compressive strength, toughness, flexural strength, and fiber–matrix bonding qualities of geopolymers based on fiber type.

### 3.1. Comparison Result for the Single Fibers with Different Material, Shape and Dimension

#### 3.1.1. Mixture Design

In this work, plain geopolymers concrete (PGPC) and three varieties of fiber-reinforced GPC were developed. The volume fractions of steel fibers (SF) and superplastic shape memory alloy fibers (Niti-SMAF) in the PGPC produced with 1.00 percent, 0.75 percent, and 0.50 percent SF and NiTi-SMA fibers were particularly interesting. Steel fibers reinforced geopolymers concrete (SFRGPC) parameters are SFRGPC100, SFRGPC75, SFRGPC50 and superplastic shape memory alloy fibers reinforced geopolymers (NiTiSMAFRGPC) parameters are NiTi-SMAFRGPC100, NiTi-SMAFRGPC75, and NiTi-SMAFRGPC50, which are the abbreviations for SFRGPC100, SFRGPC75, and NiTi-SMAFRGPC50, respectively. The PGPC included 0.20 percent, 0.15 percent, and 0.10 percent (polypropylene fibers) PPF, respectively.

#### 3.1.2. Sample Preparation and Curing Time

Fly ash, GGBFS, aggregates, and silica sand were first dry mixed for at least 3 min in an 80-liter pan mixer to verify that all ingredients were well dispersed and that all single aggregates were coated with the powder mix. Then, the alkaline solution, water, and the three admixtures were blended into the dry mixture and mixed for about 6 min until the mix was determined to be homogenous. To make a complete homogeneous paste, all ingredients are combined in a pan-mixer and mixed for around 5 min. Finally, the composite paste was poured into the mold of 100 m × 100 m × 400 mm in dimension to create prism samples for static and cyclic flexural tests and 100 m × 200 mm in dimension to create cylinder samples for compressive test and splitting tensile test. The samples were then demolded 24 h and cured in a laboratory setting (at 20 °C and 95% humidity) for a 28-day period.

#### 3.1.3. Result and Discussion

To increase the overall mechanical quality of geopolymers concrete, the researchers used reinforcement materials such as NiTi-SMAF, SF, and PPF. SF, NiTi-SMAF, and PPF were introduced into GPC at 1.00 percent, 0.75 percent, and 0.50 percent volume for SF and NiTi-SMAF, respectively, and 0.20 percent, 0.15 percent, and 0.10 percent volume for PPF, respectively. According to the research results, the mechanical properties of FRGPC increase with the addition of SF and NiTi-SMAF, but decrease with the addition of PPF. According to the findings, adding steel and NiTi-SMAF improves the mechanical properties of FRGPC. However, adding PPF decreases them. SFRGPC mixture has the highest compressive strength (39.39 MPa), split tensile strength (5.36 MPa), and flexural strength (12.53 MPa) when compared to SFRGPC and PPFRGPC mixture, while NiTi-SMAFRGPC mixture has the best cycle bending performance, with small residual deform and the most significant realignment rate in four cycles.

**Figure 2:**
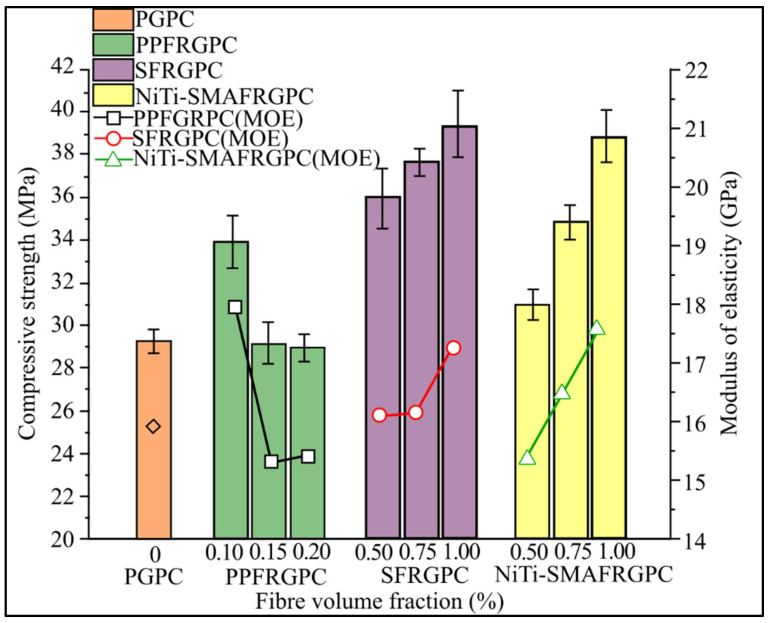
Compressive strength and MOE of PGPC, PPRGPC, SFRGPC, and NiTi SMAFRGPC under different fiber volumes [60].

Figure 2 illustrates the average compressive strength of the PGPC, PPFRGPC, SFRGPC, and NiTi-SMAFRGPC combinations. SF and NiTi-SMAF were shown to work well together to increase compressive strength. SFRGPC now has a compressive strength of 39.39 MPa, up 9.54 percent (1.00 vol percent), which is 21.50 percent greater than PCGC mixture compressive strength. The strength of compressive maximum for NiTi-SMAFRGPC has been enhanced by 20.39 percent, from 30.95 MPa (0.50 vol percent) to 38.84 MPa (1.00 vol percent). When SFRGPC and NiTi-SMAFRGPC are compared at the same fiber volume fraction, SFRGPC has a better overall value than NiTi-SMAFRGPC, but the compressive strength of NiTi-SMAFRGPC is different (25.49 percent) SFRGPC is greater than 0.50 to 1.00 volume percent (9.54 percent). SF demonstrated better axial deformation resistance than NiTi-SMAF due to their superior mechanical properties, but compressive strength increased considerably as the NiTi-SMAF concentration increased. The compressive strength of PPFRGPC, on the other hand, was observed to decrease as the amount of PPF increased. At 0.15% and 0.20% fiber volume fractions, they are even lower than PGPC, having decreased by 14.66% to 28.93 MPa (0.2 vol%) (29.16 MPa and 28.93 MPa, respectively). The contact of SF and NiTi-SMAF with geopolymers binder is stronger than that of PPF because metal material is hydrophilic, while PPF is hydrophobic; therefore, adding metal fibers to GPC can improve its mechanical properties, while a high proportion of PP fibers in GPC will reduce its mechanical properties. SF and NiTi-SMAF, as a result of their low density, tensile strength, and youthful modulus, have a higher compressive strength than PPF, but their form and size are not the same. Each size, length, and shape have unique characteristics.

Figure 2 further shows that SF and NiTi-SMAF with higher fiber content have a higher modulus of elasticity (MOE). In the 5 MPa stress–strain curve, MOE is defined as the angle between the origin and the stress of the chord. In both trials, the loading rate was adjusted at 0.5 mm/min. To obtain the stress–strain curves, a 20 mm vertical strain gauge was fitted to the compressive sample’s elastic modulus and curves. As it is less than 20% of the compressive strength of all combinations, this stress is selected. From 0.50 vol% to 1.00 vol%, the MOE values of SFRGPC and NiTi-SMAFRGPC increase from 16.13 GPa to 17.25 GPa and 15.34 GPa to 17.66 GPa, respectively. On the other hand, PPFRGPC has the highest MOE value of 0.10 vol%, then drops to 15.32 GPa, which is 0.15 vol%, and then climbs slightly to 15.40 GPa, which is 0.20 vol%. The compressive strength of the FRGPC mixture has a considerable influence on the MOE value, which is proven by the mixture of SGRGPC and NiTi-SMAFRGPC. In the case of PPFRGPC, PPF have poor mechanical characteristics leading to a decrease in compressive strength, especially at 0.15 and 0.20 vol%, the constituents of these two fibers are slightly different in terms of strength. This may alter the wire’s appearance to change the MOE and resistance trend. When comparing compressive strength trends, the MOE value achieved in the experiment is lower than that reported by other authors in prior studies, which may be due to a test scheme and management of the loading rate. Experiments revealed that SF and NiTi-SMAF can improve MOE as compared to PGPC, while a higher percentage of PPF can reduce MOE [60]. Their MOE results are not the same as the findings from previous studies, as the dimension of fibers in terms of size and form were not mentioned; however, the trend remains the same, but the result is lower than those of prior research.

**Figure 3:**
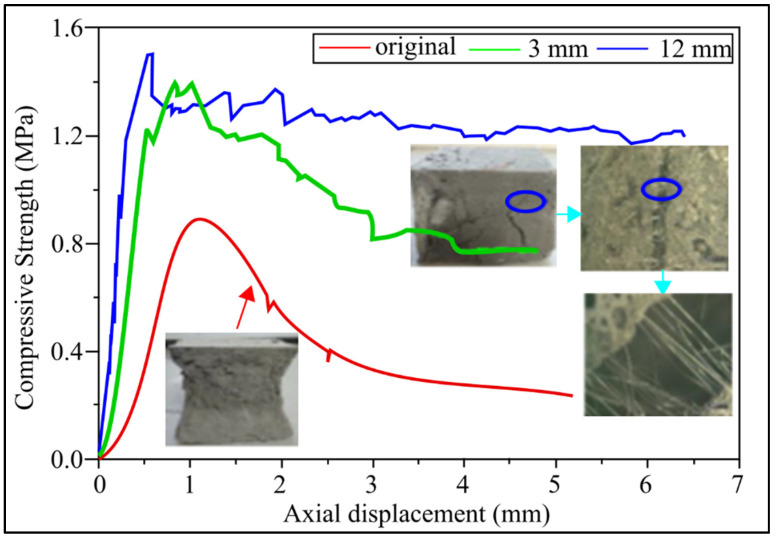
Effect of stress–displacement for different fibers length [27].

Compressive strength and MOE results are affected by the size and shape of the fibers. Figure 3 depicts the effect of PPF length on the 28-day compressive strength of lightweight geopolymers concrete. The compressive strength of fiber-reinforced fly ash-based geopolymers concrete (FLGC) with fiber lengths of 3 mm, 6 mm, 9 mm, 12 mm, and 19 mm improved by 57 percent, 46 percent, 57 percent, 71 percent, and 6 percent, respectively, as shown in Figure 3. PF samples were compared to non-PPF ones. This indicates that the length of the fibers appears to have a significant impact on compressive strength. The high strength of compressive fiber-reinforced lightweight geopolymers concrete may be due to PPF mechanically interacting with FLGC. Cracks formed on the cube sample’s surface and inside when the uniaxial load applied to it reaches its peak stress [27]. Figure 3 shows a typical observation result of a fiber-reinforced FLGC sample: the crack points in the FLGC sample are connected by fibers, reducing the number of cracks and preventing existing cracks from propagating [61]. The compressive strength of lightweight geopolymers concrete is improved by PPF.

The cracked fiber-reinforced FLGC’s residual compressive strength enables a more precise assessment of post-crack behavior. As there is no fiber–concrete link, the specimens without fibers have the lowest residual compressive strength, see Figure 3. When the axial displacement is 5 mm, the specimen’s compressive strength without PPF after failure is reduced to 25% of its peak strength. After failure under the second and third load strengths, the compressive strength of the sample with a PF of 12 mm reduces to 90 percent and 91 percent, respectively, which is roughly 1.55 times, when the axial displacement is 1.4 mm and 1.9 mm. The efficacy of fiber-free samples is at its peak, due to the bridging effect of fibers at the fracture face, which can stop the crack from progressing [62]. It can be deduced that fibers’ lengths affect the peak strength because greater fiber lengths contribute to the higher surface contact and higher displacement due to the plastic deformation. The MOE will increase the flexural strength of steel or composite materials by increasing the content of fibers [27]. However, in the polymer fibers such as PPF, the flexural strength of the material will increase with the increase in fiber content up to a certain point, and then the value will drop due to the properties (ductility and low stiffness) of the polymers [60].

When the modulus of elasticity is increased, the flexural strength is affected because with the PPF hybrid, when the fiber content is increased, the flexural strength will be opposite due to the PPF features of high elasticity and low stiffness. The qualities are influenced by the mechanism’s short and long length. Short fibers are good in an elastic condition, and long fibers are good in a plastic state; therefore, they are mixed to generate new qualities. Based on other studies, there are micro and macro cracking, with short fibers showing micro cracking and long fibers showing macro cracking. Furthermore, the effect of the fiber’s diameter on peak deflection appears to be more convoluted, which may be related to matrix characteristics and necessitates further exploration [63]. Its abnormally high deformation enhances the contact area between the fibers and matrix, improving mechanical performance in composite systems [64].

It is well known that in the case of straight fibers, the pull-out resistance is caused by two factors: the adhesion and friction combination between the mortar and the fibers. The bond area is marked by a unique linear part in a straight steel fiber bond-slip (or load deflection) curve. The debonding starts at the end of the linear segment, after which the pull-out resistance is friction. The adhesion and friction components of the mortar–fibers bond in the geopolymers (GP) mortar are higher than those of the OPC mortar. Although the fibers are made of the same material, their characteristics are also different due to differences in diameter, geometry, and shape. The type and quantity of stress generated are also fundamentally tied to the individual test undertaken, and the same behavior will not always be observed in composite materials, where numerous other elements (such as fiber orientation and group effects) are at play.

Compared with straight fibers, deformed fibers provide an additional key factor for pull resistance, namely mechanical anchoring, which allows a greater strain to be formed; as a result, compared to straight fibers, they have a higher energy absorption capacity. Despite the fact that the bonding slip behavior of deformed fibers embedded in OPC mortar has been widely studied, research on the bonding slip behavior of deformed fibers embedded in GP mortar has been limited [65].

When length-deformed fibers are pulled out, shear and tensile stress in the length-deformed area aid anchoring; nonetheless, a significant amount of local stress in the mortar may be generated. As observed with GP mortar, early failure occurs with stress surpassing mortar tensile strength. The type and quantity of stress generated are also fundamentally tied to the specific test performed, and this does not always imply that the same behavior would be observed in composite materials, which is influenced by a variety of other factors such as fiber orientation and group effects.

On the other hand, the end-deformed steel fibers exhibit higher peak bond strength at low slip, making it very suitable for enhancing the strength of interfacial bonding under low strain and deflection. The apparent frictional resistance of straight fibers to pull-out is significantly greater than that of length-deformed fibers. This means the GP in direct touch with the fibers may still be intact in the case of straight fibers, but the mortar is badly damaged or worn out in the case of fibers with deformed ends due to higher stress levels. Although PPF has a lower total pull-out resistance than trefoil fibers, trefoil fibers have a higher pull-out resistance.

Physicochemical and mechanical bonding quality are the two bonding properties found in fibers. Physical and chemical bonds are affected by the adhesion interface and friction interface, whereas mechanical bonds, as well as adhesion and friction, have an anchoring effect at the end of the fibers or along the fibers [66]. The stress transfer at a cracked section is affected by the bond properties of the fibers and matrix, and the failure behavior of the matrix varies depending on the stress distribution transferred from the fibers [67,68]. Several significant components of bonding characteristics have been investigated using bonding qualities obtained from fiber pull-out tests, including fiber shape, orientation, embedded length, surface, and matrix strength. As pre-deformed steel fibers have a substantially better binding strength than straight steel fibers, numerous researchers are considering using them as reinforcing materials. Almost all reinforcing fibers are mechanically deformed, with hook-end steel fibers being the most frequent.

Abdallah et al. [69] examined the pull-out behavior of hook-end steel fibers embedded in ultra-high-performance concrete (UHPC) in terms of fiber geometry, embedding length, and water to binder (W/B) ratio. They discovered a number of fascinating facts: (1) Lowering the W/B ratio from 0.2 to 0.11 enhances bond strength significantly; (2) the embedded length has no influence on the pull-out behavior of 5D hook fibers; (3) 5D hooked fibers are more effective than ordinary 3D and 4D hooked fibers in enhancing drawing work and strength [69]. At UHPC, Naaman and Wille compared the drawing performance of smooth and deformed (i.e., hooked and twisted) steel fibers, discovering that hooked and twisted steel fibers have a bonding strength that is four to five times that of smooth steel fibers. Wu et al. also [70] found that increasing the particle packing of the surrounding matrix can greatly improve the binding qualities of steel fibers, according to UHPC mix optimization. Stengel [69] demonstrated that roughening the surface of steel fibers using abrasion or sandpaper can improve the bonding strength of UHPC. Wu et al. [71] found that adding suitable nano-calcium carbonate (CaCO_3_) to the UHPC matrix increases the bonding strength of the interface between the straight steel fibers and the matrix by 3.2 percent. The addition of nanoCaCO_3_ to UHPC boosted straight fiber bond strength by 45 percent and drew the ability by 200 percent, respectively. Lee et al. [67] conducted several drawing tests to investigate the effect of the smooth steel fibers’ inclination angle on UHPC bonding strength, and it was discovered that the bonding strength was strongest when the fibers were inclined at 30° and 45°. On the other hand, as the tilt angle increases, the slip capacity indicates that the peak intensity slip continues to rise. In another study, Tawil and Tai [68] used different fiber types, angles of inclination, and loading rates ranging from 0.018 mm/s (quasi-static) to 1800 mm/s, to examine the impact of drawing behavior on the strength of steel fibers in UHPC. Their findings show that increasing the loading rate and inclination angle to 45 degrees, increases the pull-out resistance and energy dissipation performance of smooth steel fibers in UHPC in Lee et al. [67]. Smooth steel fibers also demonstrated the highest sensitivity to loading rate when compared to twisted and hooked steel fibers, resulting in a dynamic increase factor of 2. 32. Based on the findings of all the research, it can be concluded that fiber shape and dimension affect the properties of geopolymer-reinforced fibers, but most research only used the same material and type of fibers such as hooked and crimped. Furthermore, modification of the surface by manipulating the surface of fibers from smooth to rough to increase the surface contact area between matrix and fibers will yield better interfacial bonding; however, more study on different material with the same type of fibers, such as hooked types, is needed, as well as comparisons with other materials such as polymers of composites, because the effect type of fibers has a different effect on properties such as interfacial bonding between matrix and fibers. Table 1 summarized related previous works on the geopolymers concrete reinforcement fibers.

Wang et al. [60], in geopolymers concrete, used NiTi-SMAF, SF, and PPF as reinforcement components to improve overall mechanical characteristics (GPC). GPC includes NiTi-SMAF, SF, and PPF, with volume content of 1.00 percent, 0.75 percent, and 0.50 percent for SF and NiTi-SMAF, and 0.20 percent, 0.15 percent, and 0.10 percent for PPF, respectively. The results revealed that the mechanical characteristics of FRGPC improved as SF and NiTi-SMAF were added, but deteriorated as PPF was added. On the other hand, the high cost of NiTi-SMAF hinders broad usage in civil engineering. Due to its enticing features, it has been utilized in several specific structural infrastructures. More experimental research on NiTi-SMAF is needed for future applications, which require lightweight material by using different types of fibers of the same size and shape.

Yijiang et al. [27] investigated the thermomechanical and hygroscopic light weight properties of geopolymers concrete made using fly ash, sodium hydroxide, sodium silicate, and PPF, as well as the dry density, sodium hydroxide, PPF, aggregate, and hydrophobizing agent. Strength, thermal characteristics, and moisture absorption all played a role in their research. When the length and content of PPF are both 12 mm and 0.5 percent, the best compressive strength is achieved. In the 0–1 percent range, PPF can increase both thermal conductivity and moisture absorption. Dry density, heat conductivity, and moisture absorption are all reduced when coarse aggregate is used, with no influence on compressive strength. This research sheds light on the relationship between compressive strength and fiber size and shape. Surface-waterproofing thermal insulation materials with a high-water absorption rate, on the other hand, reduce their thermal insulation efficacy. More research is needed on different types of fibers, such as hooked or crimped, with different length parameters. It is indeed necessary to look into the impact of length displacement on hooked or crimped fibers.

Al-Majidi et al. [40] evaluated the freshness, mechanical characteristics, and microstructure of the investigated materials. The experimental results demonstrated that increasing the ground granulated blast-furnace slag (GGBS) content improved the mechanical characteristics of all investigated combinations in ordinary and steel fiber reinforced geopolymers concrete. The compression, tension, and SFRGC post-cracking cured at ambient temperature can all be improved by increasing the curing period. The discussion here is limited to steel fibers due to the large diversity of geopolymers matrix component combinations investigated in this study (2 percent volume fraction). Researchers should look into different percentages of volume fractions because different percentages of volume fractions can affect mechanical properties, microstructure properties, and the curing period.

Noishini et al. [72] conducted a comprehensive experimental program using a survey to examine the structural and material properties of synthetic fiber reinforced geopolymers concrete. This research looks at the effects of monofilament, fibrillated polypropylene fibers, and monofilament structure polyolefin fibers on the tensile and flexural properties of fly-ash-based geopolymers concrete. Macromolecular polyolefin fibers had the highest breaking energy, which could be owing to significant mechanical bonding and a low fiber aspect ratio. Models that predict the relationship between compressive and tensile strength, elastic modulus, compressive stress–strain curve, deflection, and CMOD in synthetic fiber-reinforced geopolymers concrete are created. As a result, the proposed compressive stress–strain model is acknowledged as accurately predicting the rising branch of the stress–strain curve, the strain at peak stress, and the post-peak response of ordinary and fiber-reinforced fly-ash-based GPC; however, because polymers have good ductility and low stiffness, which affects the modulus of elasticity of polymers, and because the surface of the specimen has many bubbles, the strength decreased. More studies need to be conducted to investigate why the bubbles appear at the surface of the specimen and whether it affects the strength of the specimen, the chemical reactions between polymers fibers and geopolymers, or whether the use of micro fibers increases the amount of air trapped during the geopolymer mixing.

Liu et al. [73] investigated the development of ultra-high performance geopolymers concrete (UHPGC) and the use of various SF to overcome the brittle nature of the geopolymers matrix. The researchers looked at four distinct types of straight SF with varied aspect ratios, as well as two different deformed SF. The flow capacity, compressive strength, bending behavior, including strength and deflection, and energy absorption capacity of UHPGC are all carefully assessed. SF composition boosts UHPGC’s compressive and ultimate bending strength; however, as the proportion of SF in UHPGC increases, so does its compressive and ultimate flexural strength.

Based on our review, it can be seen that from Table 1, Wang et al. [60] used NiTi-SMAF, SF, and PPF as reinforcement components in geopolymers concrete to improve overall mechanical characteristics (GPC), and the results revealed that the mechanical characteristics of FRGPC improved as SF and NiTi-SMAF are added, but deteriorated as PPF was added. On the other hand, the high cost of NiTi-SMAF hinders broad usage in civil engineering. Due to its enticing features, it has been utilized in several specific structural infrastructures. More experimental research on NiTi-SMAF is needed for future applications requiring lightweight material by using different types of fibers of the same size and shape. Yijiang et al. [27] investigated the thermomechanical and hygroscopic light weight properties of geopolymers concrete made using fly ash, sodium hydroxide, sodium silicate, and PPF, as well as the dry density, sodium hydroxide, PPF, aggregate, and hydrophobizing agent. The findings indicate that dry density, heat conductivity, and moisture absorption were reduced when coarse aggregate was used, with no influence on compressive strength. This research sheds light on the relationship between compressive strength and fiber size and shape. Surface-waterproofing thermal insulation materials with a high-water absorption rate, on the other hand, reduces their thermal insulation efficacy. More research on different type of fibers, such as hooked or crimped fibers, with different parameters of lengths is needed. Further investigations on the effect of length displacement towards hooked or crimped fibers are also required. Al-Majidi et al. [40] evaluated freshness, mechanical characteristics, and microstructure of the investigated materials. The experimental results demonstrated that increasing the GGBS content improved the mechanical characteristics of all investigated combinations in ordinary and SF reinforced geopolymers concrete. The compression, tension, and SFRGC post-cracking cured at ambient temperature can all be improved by increasing the curing period. The researchers should investigate the various types of the percentage of volume fraction to obtain various mechanical properties, microstructure properties, and the effect curing period based on volume fraction percentage. Noishini et al. [72] conducted a comprehensive experimental program using a survey to examine the structural and material properties of synthetic fiber reinforced geopolymers concrete. As a result, the proposed compressive stress–strain model is acknowledged as accurately predicting the rising branch of the stress–strain curve, the strain at peak stress, and the post-peak response of ordinary and fiber-reinforced fly ash-based GPC; however, the strength decreased because polymers have good ductility and low stiffness that affect the modulus of elasticity of polymers and many bubbles appeared at the surface of specimen. More studies need to be conducted to investigate why the bubbles appear at the surface of specimen and whether it affects the strength of specimen, the chemical reactions between polymers fibers and geopolymers or whether the use of micro fibers increases the amount of air trapped during the geopolymer mixing. Liu et al. [73] investigated the development of UHPGC and the use of various steel fibers to overcome the brittle nature of the geopolymers matrix. The flow capacity, compressive strength, bending behavior, including strength and deflection, and energy absorption capacity of UHPGC are all carefully assessed. Steel fiber composition boosts UHPGC’s compressive and ultimate bending strength; however, as the proportion of steel fibers in UHPGC increases, so does its compressive and ultimate flexural strength.

### 3.2. Comparison Results of the Hybrid Fibers with Different Material, Shape, and Dimension

After steel and polypropylene fibers were hybridized, the bending capabilities of fiber-reinforced geopolymers were investigated. Geopolymers’ brittleness has been improved by adding fiber reinforcement. There are numerous types of fibers available nowadays. PPF lose strength rapidly and have a reduced post-peak response after the first rupture due to their high flexibility and low stiffness. To solve these issues, a hybrid system based on high-strength and stiff fibers (such as SF) has been created. Replacement and addition are the two types of hybrid systems. In the replacement system, PPF is replaced by SF gradually at a rate of 0.2 percent by volume, while in the addition system, steel fibers are added to the mixture at a steady rate. According to the findings of both of the hybrid systems (replacement and addition), SF hybridization can improve the bending response, toughness, and residual strength of PPR reinforced geopolymers to varying degrees [5,65]. The load reduction, as well as the second peak, appeared to improve almost instantly. Hardness and residual strength continuously rise as the number of steel fibers in the mix increases [5]. The goal of this study is to develop a novel fiber system by combining two types of materials without using composites. If a composite process is being used, this procedure can minimize the cost of using fiber and the cost of the process by combining different types of fibers or using the same material but with different features such as shape and dimension.

**Figure 4:**
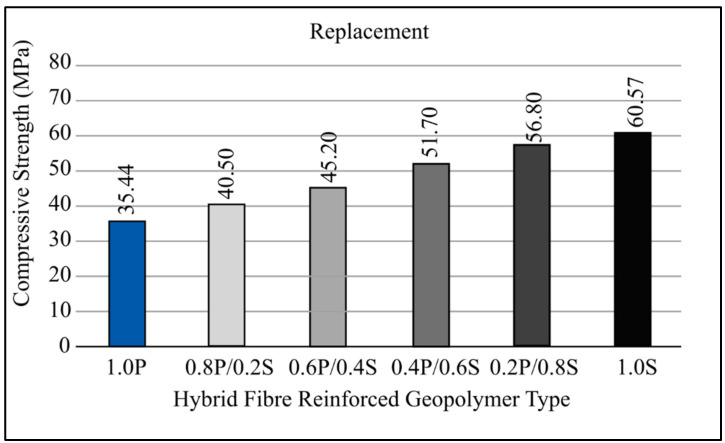
Compressive strength of r-HyFRG [5].

Figure 4 depicts the compressive strength measurements of a single variety of fiber-reinforced geopolymers (FRG). Ordinary geopolymers have a compressive strength of around 40 MPa. Steel fibers’ compressive strength improves to around 56.6 and 61.7 MPa for 0.5 percent and 1.0 percent volume fractions, respectively. Steel fibers’ compressive strength rises as the number of fibers in the material rises.

The compressive strength of 0.5 percent PFRG was found to be greater than that of traditional geopolymers. The considerable increase in compressive strength of the two types of FRG is because of silica fume added to the geopolymers mixture, which can strengthen the link between the fibers and the geopolymers matrix. This observation is in line with the findings of a previous study by Al-Majidi et al. [40]. According to their findings, adding 10% non-densified silica fume to SFRG increases compressive strength significantly. The compressive strength of PFRG declined dramatically from 47 MPa to 35 MPa when the fraction of fibers grew to 1%. The cause is thought to be a lack of compaction and significant voids inside the material. PPF is a versatile material. Compaction becomes problematic when the volume percentage is considerable, causing the geopolymers matrix to become loose and porous.

When the base fibers (1% PPF) are changed to SF in the hybrid FRG substitution type (r-HyFRG), the volume fraction increases by 0.2 percent. The results reveal that compressive strength rises as the fraction of SF rises. The strength of the mixed FRG increased quickly after the SF were added. This hybrid FRG has a maximum strength of 56.8 MPa and is made up of 0.2 percent PPF and 0.8 percent SF. All r-HyFRG samples, however, have a lower compressive strength than 1.0 percent steel FRG. Figure 4 depicts the result of adding steel fibers to a base of 1.0 percent PFRG, which was enhanced to 1.0 percent (in 0.2 percent increments) by adding hybrid FRG in 0.2 percent increments (a-HyFRG). Similar to alternatives, a compressive HyFRG’s strength improves as the number of SF increases.

Figure 4 shows a comparison of three results and concludes that compressive strength is the best. Identical fibers were employed, but the mixed design approach is different (replacement and addition). Although both graphs indicated an upward trend, there are advantages and disadvantages when it comes to cost and weight. Figure 4 shows how using a mix design replacement can minimize the weight of GPC and the amount of geopolymers-based material utilized.

The percentage of load drop is also affected by fibers type and content. Due to the high flexibility and low stiffness of polypropylene FRG fibers, a significant drop in load is often observed. According to the reports, the maximum drop is about 75% for every 0.5% of PPFRG. As the fibers content increases, the proportion of decline decreases. Steel fibers can increase load faster than polypropylene fibers due to their strong strength and rigidity.

**Figure 5:**
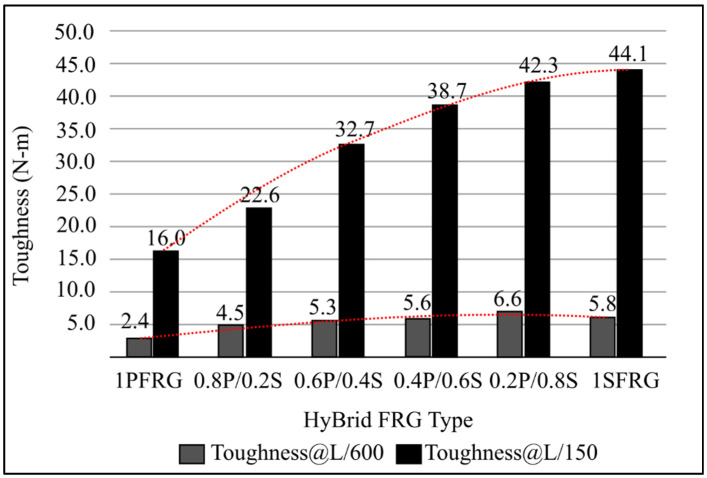
Toughness of r-HyFRG [5].

According to ASTM C1609, toughness and residual strength are computed. Toughness is defined as the area under the load deflection curve, which reflects how much energy the specimen can withstand. At two different deflections, L/600 and L/150, two toughness values are calculated in general. The equivalent flexural strength of the specimen after initial cracking is expressed as a percentage of its original strength. Figure 5 depicts the research findings during the initial period of high demand.

Figure 5 shows the results of the HyFRG toughness test. The toughness of hybrids increased with the degree of replacement or addition, regardless of the hybrid type SF, despite the fact that the rate of growth is slowing down with the increase in SF when it comes to the r-HyFRG. The toughness of a big deflection (L/150) never exceeds the toughness of a small deflection (L/100) because the total fiber volume fraction is regulated at 1.0 percent, 1SFRG has a value of 1. In the case of a-HyFRG, however, an increase in total fiber content has a distinct effect on flexural performance. SF was added to the mix. Although the conversion of fiber into a lower amount of fiber is expected to improve the hardness, the greater the difficulty, a non-uniform distribution of the fibers in the mix can have a negative impact on the FRG’s performance.

According to Figure 5 of the bending response, the higher the first peak load, the smaller the percentage of load decrease, and the larger the following (second) peak. In terms of bending performance, it has been demonstrated that increasing the number of steel fibers in the mix improves both toughness and equivalent bending strength [5]. A comparison of replacement and addition for the same fiber content shows that the substitution method appears to generate better results than the addition system. Furthermore, when total fiber content exceeds 1% by volume, the advantages appear to diminish. High fibers concentration makes mixing difficult, resulting in poor compaction, uneven fibers dispersion, and increased void volume [34]. Although a hybrid system can address some of PFRG’s flaws, caution should be exercised when choosing a hybrid system and overall fiber content. By comparing single and hybrid fibers, good results may be obtained, and the problem of fiber weakness due to compressive, flexural, or toughness in geopolymers reinforced fibers can be resolved. The next section discusses the bonding between fibers and matrix at geopolymers concrete reinforced fibers.

### 3.3. Comparison of Interfacial Bonding between Matrix and Fibers Due to Type of the Materials

The bonding of GPC and fibers in geopolymers concrete reinforced fibers will be discussed in this part. Fibers have been widely used in the GPC to address the problem of brittleness and low toughness in the GPC.

The mechanical and microstructural properties of geopolymers made from fly ash and reinforced with three distinct types of fibers were investigated in prior research. The effects of adding SF, PPF, and polyvinyl alcohol fibers (PVAF) to geopolymers composites on strength, wear resistance, and drying shrinkage were investigated. A microstructure analysis was also carried out to better understand the composition of the geopolymers matrix and its relationship with the fibers. SF and PVAF boosted the geopolymers composite’s bending strength by 31.45 percent and 39.84 percent, respectively, as compared to the control sample. Drying shrinkage of fewer than 400 microstrains and abrasion resistance of less than 1 gram are also features of all fiber-reinforced geopolymers composites. The non-fibers geopolymers control sample has a high degree of geopolymerization, while the fibers and geopolymers binder have adequate interface bonding, according to microstructure examination [2]. 

Previous studies have been investigating the bending behavior of geopolymers composites composed of fly ash and reinforced with SF and PPF. Thermal curing improves the strength properties of coarse fibers reinforced geopolymers composites; however, the compressive strength of PPF does not increase significantly, but it does show some improvement in indirect tensile and flexural strength [34].

**Figure 6:**
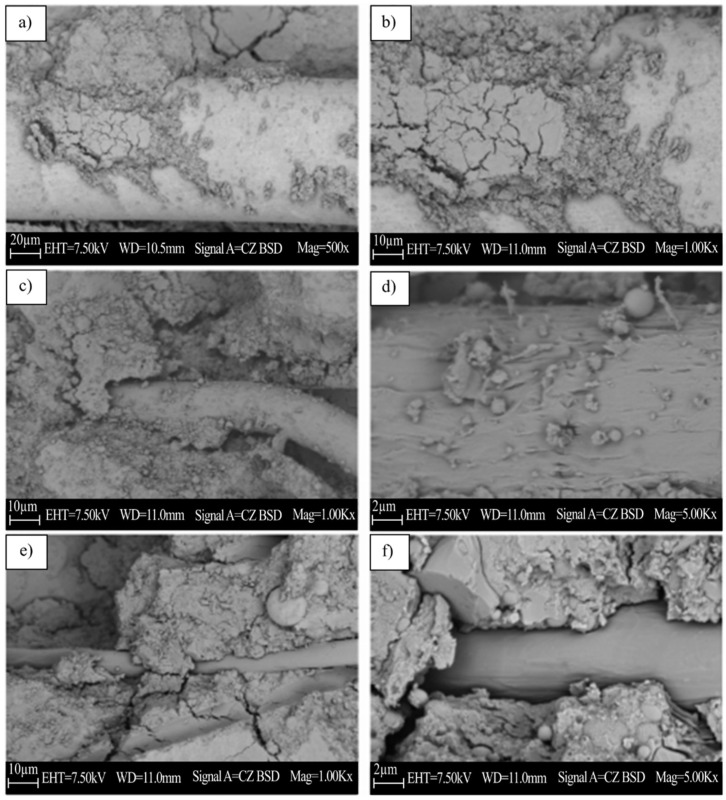
SEM micrographs the investigated (**a**,**b**) steel fibers sample, (**c**,**d**) polyvinyl alcohol fibers sample, (**e**,**f**) polypropylene fibers sample [2].

Figure 6 shows the SEM micrographs of non-fibers and fiber-reinforced geopolymers samples. In the compressive strength test, a sample is collected from the portion of the failed material adjacent to the failed surface. According to reports, some unreacted fly ash particles can be seen in [74]. The strength behavior under compression is positively correlated with unreacted fly ash particles. Figure 6a,b show the micrographs of steel-reinforced geopolymers composites magnified at different magnifications. It can be inferred that the presence of fibers leads to a high degree of adhesion, and there is no visible deformation on the surface of the fiber, which is an ideal characteristic in terms of durability and long-term quality. According to the conclusion by Xu et al. [75], based on the SEM observation of the PVAF reinforced mortar, the photomicrograph exhibited the thin layer of geopolymers produced during the geopolymerization reaction. This discovery indicates that the polyvinyl alcohol fibers and the geopolymers matrix have a good level of bonding. Figure 6e,f show the smooth surface of the fibers and the space where the fibers are pulled out in the polypropylene sample. The geometry of the fibers explains why PP fibers perform poorly in comparison to other fiber types; in addition, the blank area clearly demonstrates debonding between the fibers and the matrix. This outcome is consistent with Ranjbar et al.’s [21] earlier studies. According to the research, such failures are related to the poor performance of PPF reinforced geopolymers. This study provides a useful comparison. The microstructure inspection of the control sample showed that there is good geological polymerization between the fly ash and the alkaline solution. Both SF and PVAF are made into composite materials with good internal characteristics and acceptable fibers and geopolymers matrix interface bonding. On the other hand, the photomicrograph of PPF shows that its performance level is lower compared to other fibers. Compressive strength, toughness, and flexural strength are all affected by the interface bond between fibers and GPC.

The use of previous reinforcing geopolymers materials with fibers is another technique to improve tensile strength, flexural strength, and fracture toughness. When attempting to increase the performance of a fiber-reinforced composite, the link between the fibers and the geopolymers matrix is a significant consideration. SF and PVAF implanted in geopolymers matrices are tested for single fiber pull-out, using OPC mortars as a control. Fiber type and shape (for example, steel and polypropylene), alkali solution concentration in the geopolymers matrix, and curing conditions are all important factors to consider. In comparing and resolving the adhesive slip performance, failure mode, and anti-slip process of different substrates and fibers, fiber deformation rate is a new approach of quantifying the impact of fiber shape on mortar performance. In the case of SF, the geopolymer–fiber composite material outperforms the complete fibers drawing mechanism at a lower deformation rate of the fibers. Because of the great bearing capacity and high adhesion strength of the geopolymers SF interface, a higher deformation rate will result in brittle failure properties, such as fiber fracture or matrix failure [65]. The outcome of this study is positive since it performed a thorough examination of the bonding fibers and matrix, which was based on the dimensions, shape, and material of the fibers. Even though they are made of the same substance, each fiber has its unique set of characteristics.

In addition, direct chemical bonds were observed at the interface of geopolymeric steel [76]. This is largely responsible for the tight bond between these two materials. The GP mortar’s strong adherence to SF and PPF straight fibers could be attributable to the denser interfacial transition zone (ITZ) between the aggregate and the GP paste. Steel fiber reinforced GP concrete showed the same tendency, according to Sarker et al. [77]. Another reason could be that fly-ash-based GP mortar has a lower drying shrinkage than OPC mortar, preserving the interface area around the fibers, as demonstrated in the scanning electron microscope image of the mortar–fibers interface (SEM, Figure 6). More research is needed to better understand the various chemical and physical mechanisms that occur during the ITZ between GP and fibers.

The peak bond, pull-out, and equivalent bond strength of single fibers are commonly determined using a single fiber pull-out test. To characterize the performance of fiber interfaces and mortar interfaces in composite based on reinforced cement [65]. Another reason could be that fly-ash-based GP mortar has a lower drying shrinkage than OPC mortar, preserving the interface area around the fibers. To better understand the many chemical and physical mechanisms involved in the ITZ between GP and fibers, more research is needed.

**Figure 7:**
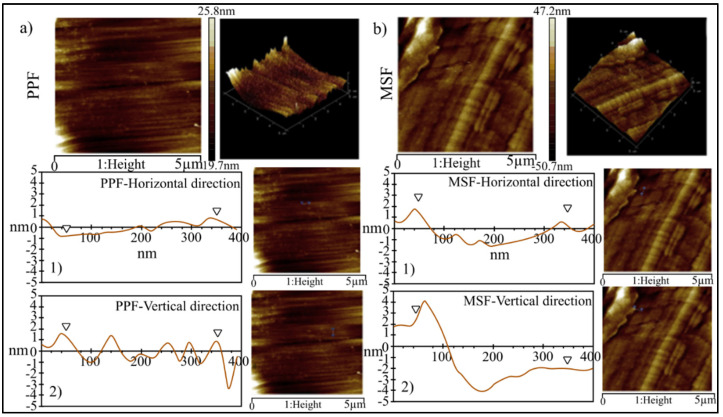
PPF (**a**) and MSF (**b**) surface observation a nanometric scale using AFM [21].

The surface topography images of PPF and micro steel fibers (MSF) are shown in Figure 7a,b. The PPF has a basic structure consisting of 50–75 nm thick grooves oriented along the vertical axis of the fibers, as shown in the 3-D model (Figure 7). The height of these grooves was determined by determining the surface profile perpendicular to the fiber’s axis, and it was found to be approximately 2 nm. In addition, the atomic force microscopy (AFM) photograph reveals the secondary structure of the surface of the fiber, which is superimposed on the main vertical grooves, which is made up of tiny grooves and grains that run parallel to the horizontal axis of the fibers but are not visible in the field emission scanning electron microscopy (FESEM) image. On the other hand, the surface of the steel fibers has a completely different appearance, consisting of a rather rough structure formed by the SF.

**Figure 8:**
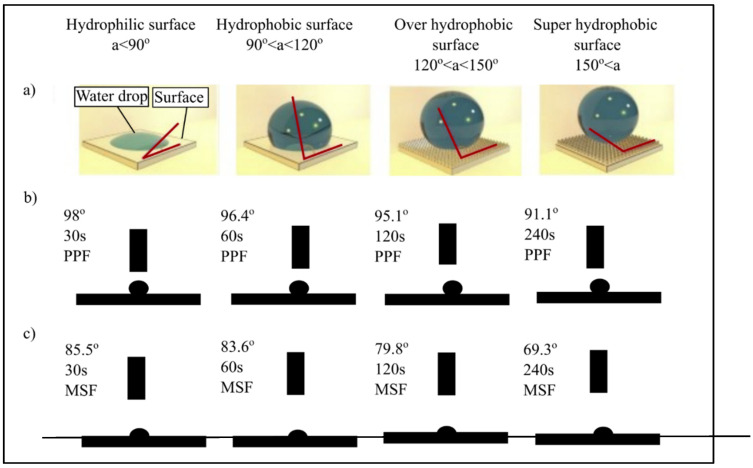
The general categorization of hydrophobicity (**a**) and water contact angle of PPF (**b**) and MSF (**c**) at 30, 60, 120, and 240 s [21].

The bonding between matrix and reinforced geopolymers can determine the efficiency of reinforcement in a composite. Geopolymers are water-based; if the fibers are wettable in water, there is a lot of potential for a good link between the reinforced geopolymer and the matrix [78]. The apparent contact angle is the primary indicator of the material’s wetting capabilities. Materials are classified as hydrophilic or hydrophobic depending on whether the contact angle is less than or larger than 90 degrees, which indicates whether they absorb or repel water. Figure 8a shows how materials are classified according to their contact angle qualities [79]. Figure 8b,c show PPF and MSF angle of water contact, which were used to determine their wettability. Polymers are often hydrophobic, resulting in low surface energy and a water contact angle of greater than 85 degrees [80]. The contact angle, which will reflect the bonding between fibers and matrix, is the reason for the comparison between PPF and MSF fibers. As a result, by understanding the properties of each material’s contact angle, you can choose the type of fibers, shape, or geometry that will eliminate or reduce the problem of fiber contact angle.

The impact of hydrophobicity and matrix shrinkage in geopolymers on short- and long-term fiber–matrix interactions in related composites are discussed. Due to its non-polar C-C bond and short-distance ripple roughness, PPF behaves as a hydrophobic material that repels water [81]. As a result, geopolymers have poor interface contact with PPF in their fresh condition as a water-based matrix, and air bubbles are trapped between the corrugations on the fiber’s surface and the matrix paste; however, as the geopolymers shrink over time, internal tension will develop in the composite material. The effect of shrinkage causes the fibers to debone and expand the distance between the two phases because of the weak contact at the matrix–fibers interface. In comparison to PPF and MSF are hydrophilic, allowing water to diffuse across their surface. The hydrophilicity of MSF, together with grooves located at a large distance from the surface (as mentioned in Section 3), results in a firm contact between the MSF and the substrate in the fresh phase. The combination of the oxide layer and the copper coating on the steel fibers leads to a more hydrophilic surface, resulting in a stronger bond between the MSF and the substrate [76]. The strong interface contacts between MSF and the geopolymers matrix, as well as the greater Young’s modulus of the fiber, led to shrinkage control and improved the mechanical qualities of the related composite through lower fiber inclusion when compared to PPF. Although individual thread toughening techniques are negligible, a large number of fibers spread out over a long distance can greatly contribute to the composite’s energy absorption [82]. According to our findings, fiber wettability, material type, and roughness all have a role in matrix and fiber interaction, with a material with a high wettability and a corrugated surface having the best results. A hydrophobic fiber with a smooth surface made stronger contact with the geopolymers binder; yet, the binder’s future development may be influenced by shrinking long-term strength; as a result, these two aspects are accompanied by one another and must be taken into account while evaluating the fiber geopolymers composites with reinforcement.

MSF added to a fly ash-based geopolymers matrix can greatly improve energy absorption, flexural strength, and shrinkage reduction without compromising compressive strength. On the other hand, PPF has weak contact with the surface due to its hydrophobicity. The adhesive is in its initial form and then debones from the substrate over time. Therefore, the addition of PPF leads to a decrease in bending stiffness and strength; however, energy absorption is improved when compared to geopolymers paste without fibers.

**Figure 9:**
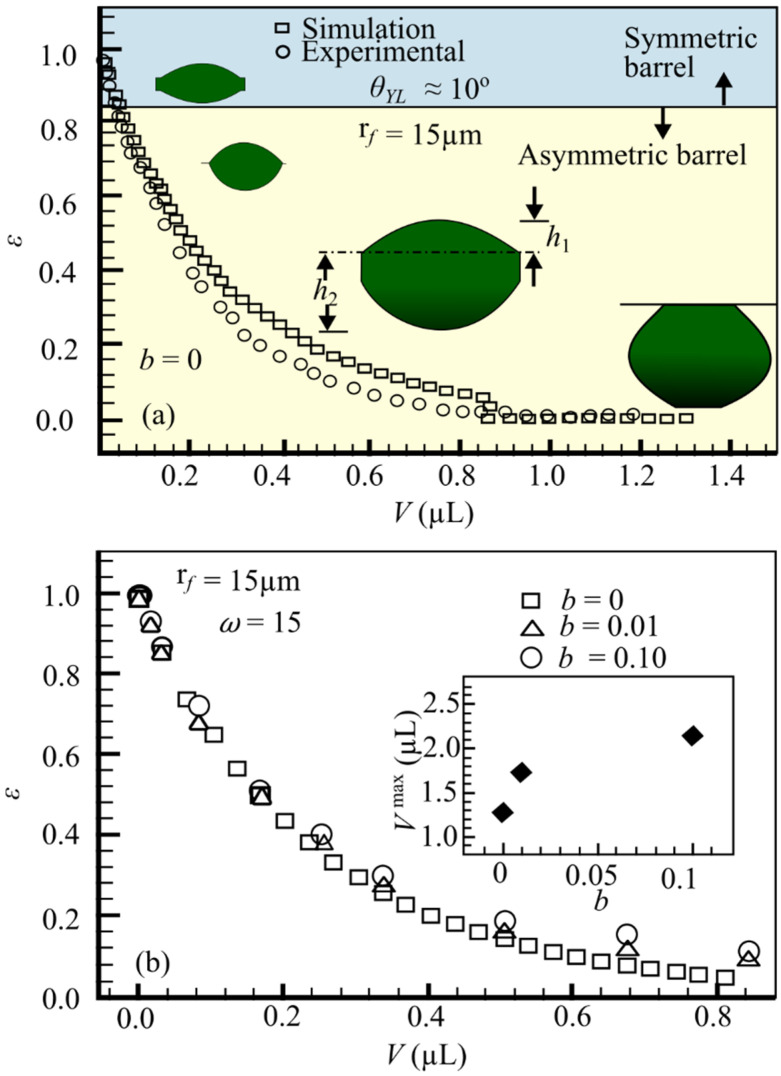
The droplet volume of ultra-low sulfur diesel (ULSD) droplets on smooth PP fibers is compared to the asymmetry factors from experiments and computer simulations (**a**). The asymmetry factor for the radius is indicated in (**b**). On the same fibers but with varying roughness amplitudes, the inset indicates the greatest droplet volume possible [59].

Figure 9 shows the impact of fiber roughness on droplet shape for fibers with a diameter of $r_{f}=15\;\mu m$, a roughness frequency ω=15, and a YLCA of $\theta_{\mathrm{YL}}=30{}^{\circ}$, but varied fiber roughness amplitudes of b = 0, 0.01, and 0.1. According to these findings, the transition from a symmetric to an asymmetric barrel form occurs at a greater droplet volume for rougher fibers. In other words, when the fiber is rougher, a barrel-shaped droplet has a higher likelihood of remaining symmetric. The greatest droplet volume can stay connected to fibers under gravity.

As the roughness of the fibers rises, so does the maximum droplet volume. The Wenzel equation predicts that these results will occur: When a smooth surface is roughened, it becomes more “philic” [83]. This novel method enabled the use of a wide range of fluids on “philic” and “phobic” fibers, as well as the development of an empirical relationship between droplet detachment force from fibers, droplet contact-line length, and fluid surface tension (but not droplet size or its contact angle with the fibers). As illustrated in Figure 9, the contact angle is regulated by the surface roughness; the rougher the surface, the better the contact and bonding between fibers and matrix.

Simulating the failure response of carbon fiber reinforced polymers (CFRP) necessitates the use of proper constitutive damage models for the matrix and fiber phases, as well as their interfaces, in addition to reconstructing the microstructure and generating a conforming mesh [84]. Fiber–matrix debonding has been found to be one of the key damage mechanisms influencing mechanical behavior when macroscopic stresses are applied transverse to the fiber’s direction [85,86]. Failure is dominated by the production of shear bands in the matrix under transverse compression and shear pressures. When applying loadings longitudinally along the fibers’ direction, it is also important to consider fiber fracture and kink-band development as potential damage mechanisms [83].

Ranjbar et al. [21] investigated the effects of shrinkage of the fly-ash-based geopolymers binder on the wettability, chemical properties, and nano-roughness of two distinct fibers; PPF and MSF, superimposed on the fibers–matrix. The interaction is created, as well as the mechanical properties that go along with it. MSF added to the fly-ash-based geopolymers matrix enhanced energy absorption, lowered bending strength, and reduced shrinkage, but had no influence on compressive strength. Due to the fact that PPF is hydrophobic, it makes a poor initial contact with the adhesive and eventually debonds from the substrate. As a result, more research into the various sizes and shapes is required.

Amrei et al. [59] examined how surface roughness affects the equilibrium form and apparent contact angle of droplets deposited on fibers. The droplet morphology on rough fibers was investigated using the energy minimization method used in the surface evolver finite element system. In contrast to past publications, the data are summarized in a phase diagram. As the roughness of the fibers rises, the apparent contact angle decreases, but the effect diminishes as the droplet size grows larger in comparison to the roughness amplitude or frequency; however, as the droplet volume grows, separation force roughness has less of an impact. It is indeed necessary to look into different types of roughness and fiber materials in order to calculate the droplet volume growth rate as a function of the roughness size of the fiber surface.

Rolf et al. [51] studied various configurations of fibers types, embedded lengths, and angles through laboratory tests and analysis models. A laboratory test of fiber pull-out is performed to study the fibers–matrix bonding mechanism. Fibers form, tensile strength, concrete strength, and fiber inclination are all factors that affect the pull-out reaction of the fibers. Based on the experimental results, the effects of these factors on the relationship between pull-out force and displacement, fiber efficiency, and fiber or matrix failure reaction are investigated. Straight fibers have lower tensile strength than other types. The strong anchoring in high-strength concrete causes crimping and biconical fibers to rupture. Hooked-end fibers, on the other hand, pull fully out of the way and are more flexible. However, straight fibers are angled in relation to the loading direction, and misalignment of the fibers adds to the complexity. Further study needs to be conducted to examine other types of fibers to obtain a better understanding of the relationship between angle and length of fibers towards fracture stress.

Kim et al. [69] examined the influence of fiber type and spacing between fibers on the pull-out behavior of steel fibers embedded in UHPC. To this aim, three distinct types of steel fibers were evaluated, namely straight, hooked, and twisted, as well as four various fiber spacings, which corresponded to fiber volume fractions of 1%, 2%, and 7%, and a fiber bundle. Four distinct fibers were included in a single dog bone sample to investigate the influence of the distance between the fibers, and a single fibers sample was also generated as a control sample for testing. The twisted fibers in the UHPC matrix obtained the highest bond strength, which is approximately greater than the bond strength of straight fibers and hook fibers. It is agreed that the twisted shape has a strong bonding force between the fibers and the matrix because it causes greater damage to the matrix due to the key interface of the bond during the pull-out test. More study is required using other materials of fibers that have hydrophobic properties; therefore, it can be useful data if it works on other materials of fibers that have poor interfacial bonding between matrix and fibers.

Bhutta et al. [65] posited that the bond between the fibers and the geopolymer matrix is an important issue to investigate in order to improve the performance of fiber-reinforced composites. SF and PPF implanted in a geopolymers matrix were subjected to single fiber pull-out tests, with OPC mortar serving as a control condition. The fracture of GP mortar reinforced with length-deformed steel fibers before it is entirely taken out does not represent fibers due to the brittleness of GP mortar and significant fiber deformation; however, under different steel fibers deformation rates, the GP matrix performs better at a lower RD value. The main resistance mechanism of fiber–mortar adhesion, which is influenced by the morphology and quality of the fiber–mortar interface, needs to be investigated.

From our review, it can be seen that from Table 2, Ranjbar et al. [21] had investigated the effects of shrinkage of the fly-ash-based geopolymers binder on the wettability, chemical properties, and nano-roughness of two distinct fibers; PPF and MSF, superimposed on the fiber–matrix. The interaction is created, as well as the mechanical properties that go along with it. MSF added to the fly-ash-based geopolymers matrix enhanced energy absorption, lowered bending strength, and reduced shrinkage but had no influence on compressive strength. PPF has hydrophobic qualities, resulting in weak initial contact with the adhesive and eventually debonding from the substrate. As a result, the various sizes and shapes must be investigated further. Amrei et al. [59] examined how surface roughness affects the equilibrium formed and apparent contact angle of droplets deposited on fibers. As the roughness of the fibers rises, the apparent contact angle decreases, but the effect diminishes as the droplet size grows larger in comparison to the roughness amplitude or frequency; however, as the droplet volume grows greater, separation force roughness has less of an impact. It is indeed necessary to look into different types of roughness and fiber materials in order to calculate the droplet volume growth rate as a function of the roughness size of the fiber surface. Rolf et al. [51] studied various configurations of fibers types, embedded lengths, and angles through laboratory tests and analysis models. A laboratory test of fiber pull-out is performed to study the fiber–matrix bonding mechanism. Based on the experimental results, the effects of these factors on the relationship between pull-out force and displacement, fiber efficiency, and fiber or matrix failure reaction are investigated. Straight fibers have lower tensile strength than other types. The strong anchoring in high-strength concrete causes crimping and biconical fibers to rupture. Hooked-end fibers, on the other hand, pull fully out of the way and are more flexible; however, straight fibers are angled in relation to the loading direction, and misalignment of the fibers adds to the complexity. More research is needed for other types of fibers to better understand the relationship between angle and length of fibers in relation to fracture stress. Kim et al. [69] examined the influence of fiber type and spacing between fibers on the pull-out behavior of steel fibers embedded in UHPC. The twisted fibers in the UHPC matrix obtained the highest bond strength, which is approximately greater than the bond strength of straight fibers and hook fibers. It is agreed that the twisted shape has a strong bonding force between the fibers and the matrix, because it causes greater damage to the matrix due to the key interface of the bond during the pull-out test. More research is needed with other fibers that have hydrophobic properties. Therefore, it can be useful data if it works on other materials of fibers that have poor interfacial bonding between matrix and fibers. Bhutta et al. [65] posited that the bond between the fibers and the geopolymer matrix is an important issue to investigate in order to improve the performance of fiber-reinforced composites. The fracture of GP mortar reinforced with length-deformed steel fibers before it is entirely taken out does not represent fibers due to the brittleness of GP mortar and significant fiber deformation; however, under different steel fibers deformation rates, the GP matrix performs better at a lower RD value. The main resistance mechanism of fiber–mortar adhesion, which is influenced by the morphology and quality of the fiber–mortar interface, needs to be investigated.

## 4. Summary and Future Works

From our review, a few things can be concluded. Firstly, fiber-reinforced geopolymers have superior compressive strength and toughness properties. This was due to the brittle properties of geopolymers. Steel, polymers, and composite fibers are among the many types of fibers available. Each type of fiber has its own characteristics that influence the bonding between matrix and fibers.

Further, the factors that affect the geopolymer’s properties have been reviewed in this paper. There are two factors that contribute to the superior qualities of geopolymer-reinforced fibers. Fibers of various shapes and dimensions, such as hook-end, straight, crimped, and others, each have their own characteristics that affect the properties of geopolymer-reinforced fibers. Long and short fibers, for example, are good for toughness and short fibers are good for strength, but the shape also affects the strength between matrix fibers. Microfibers can control micro-crack propagation because the energy transfer between matrix fibers is lower, according to research.

Furthermore, reviews have also been published on the geopolymer-reinforced fiber’s properties. The material of fiber contributes to the bonding strength between matrix and fibers. Steel fibers have good bonding between matrix fibers compared to polymers fibers, since steel fibers have good interface bonding between matrix and fibers due to the hydrophilic surface properties (contact area angle of surface between matrix and fibers); however, roughness and smoothness also affect the interfacial bonding between matrix and fiber. Mostly roughness surface of fibers has good bonding compared to the smooth surface of fibers.

Moreover, this paper also reviewed the lightweight geopolymers reinforced polymers fibers. The compressive and toughness strength were improved. The fibers can help to prevent cracking and a loss of compressive strength. Hence, adding the fibers improved the properties of the geopolymer-reinforced fibers.

Based on our review, a few gaps have been identified, and several future works are proposed in this study as listed below:

- More study on the shape of the fiber is required as most fibers are round in shape. Creating new shapes such as diamonds and rectangles will result in a different outcome.
- Modification or chemical treatment of fibers surface which has hydrophobic properties.
- Further investigation on sustaining the properties of geopolymers reinforced fibers at different temperatures.
- Studies on hybrid fibers have yielded positive results, primarily with steel and polymers or a combination of two types of fibers, but more work is needed with other materials and combinations of more than two fibers.
- The geopolymer properties need to be observed based on the mechanical and physical properties depending on the desired application, such as lightweight, green product, low cost, and high impact.
- Research on the thermal conductivity of geopolymer-reinforced fibers.

## Figures and Tables

**Table 1:** Research on geopolymers concrete reinforced fibers.

No	Author	parameter	Variable	Properties	Material Geopolymers	Material Fibers and Shape	Findings
1.	Wang et al. [60]	PPFRGPC 0.10%,0.15%,0.20% by volume. SFRGPC and NiTi-SMAFRGP 0.50%,0.75%,1.0% by volume.	The two are loaded at a rate of 0.5 mm per minute. Compressive strength of 20 mm. A 0.5 mm/min loading rate was used in the static flexural test.	Compressive test splitting tensile	Fly ash blast furnace slag	NiTi shape memory alloy (half-circle hooked ends) steel (hooked ends) polypropylene fibers (crimped)	NiTi-SMA fibers outperform steel and PP fibers in terms of compressive strength, splitting strength, elastic modulus, and static bending strength, as well as cyclic bending performance.
2.	Yijiang et al. [27]	The fibers lengths of 3 mm, 6 mm, 9 mm, 12 mm and 19	Wet density Dry density	Compressive test	Fly ash	Polypropylene fibers	Fiber-reinforced FLGCs with fiber lengths of 3 mm, 6 mm, 9 mm, 12 mm, and 19 mm improved compressive strength by 57%, 46%, 57%, 71%, and 6%, respectively.
3.	Al-Majidi et al. [40]	Particle size (d (0.5)), and d (0.1) and d (0.9). Steel fiber was added at 2% volume fraction.	Curing time, specifically after 3, 7, 14 and 28 days. Compressive cubic 50mm and tensile dog bone shape.	Compressive test direct tensile	Fly ash category (S) GGBS SF	Steel fibers	When the curing time of SFRGC treated at room temperature was doubled, the compressive, tensile, and post-cracking behaviour of the material improved dramatically.
4.	Noushini et al. [72]	Polypropylene (PP) fibers, 18 mm monofilament, 19 and 51 mm fibrillated PP fibers, and 48 and 55 mm embossed polyolefin (PO) fibers are all available.	cylinders (100 mm × 200 mm). prisms (100 mm × 100 mm × 550 mm). prims (150 mm × 150 mm × 600 mm).	Compressive test Flexural test	Fly ash slag	Polypropylene fibers Polyolefin	Compared with ordinary GPC, the compressive strength of FRGPC containing polypropylene fibers is reduced by 1–7% on average. Despite the slight decrease in strength, the bending performance has been significantly improved. Polyolefin blends also caused the greatest improvement in fracture energy.
5.	Liu et al. [73]	0%, 1%, 2%, and 3% by volume of concrete (vol%) were used.	steam curing at 80 °C standard curing at 20 °C	Compressive test Flexural test	Fly ash class F Silica fume	Steel fibers (straight, hooked-end, corrugated)	The compressive and ultimate bending strength of UHPGC increases with the increase in steel fibers content.

**Table 2:** Research on the bonding between matrix and fiber.

No	Author	Parameter	Properties	Material	Material of Fibers and Shape	Findings
1.	Ranjbar et al. [21]	The number of fibers in geopolymers paste ranged from 0.5 percent to 1 percent, 2 percent to 3 percent, and 4 percent by volume	AFM FESEM	Low calcium fly ash.	Micro steel Polypropylene.	MSF improves energy absorption, reduces bending strength, and shrinkage when added to a fly-ash-based geopolymers matrix, but has no influence on compressive strength. PPF has hydrophobic qualities, resulting in weak initial contact with the adhesive and eventual debonding from the substrate.
2.	Amrei et al. [59]	-	Propylene Glycol (PG) Ultra-Low Sulfur Diesel (ULSD).	-	Polypropylene.	The finding is not in line with the previous research. When the droplet size grows with respect to the roughness amplitude or frequency, the apparent contact angle falls, but the effect becomes less significant.
3.	Rolf et al. [51]	Fibers shape. Fibers tensile. Strength. Concrete strength. Inclination angle.	Pullout test.	OPC	Steel (hooked end, crimped, twin cone).	Non-straight fibers outperformed straight fibers in terms of pullout resistance and fiber efficiency. Crimped and twin cone fibers ruptured as a result of strong anchorage in high-strength concrete. Hooked-end fibers, on the other hand, demonstrated complete pullout and a more ductile reaction. extra difficulties imposed by fiber misalignment; straight fibers inclined with respect to the loading direction.
4.	Kim et al. [69]	Fibers volume fractions of 1%, 2%, and 7%.	Pullout test	OPC Silica fume	Steel (straight, hooked end, triangle).	The twisted fibers in the UHPC matrix obtain the highest bond strength, which is approximately greater than the bond strength of straight fibers and hook fibers.
5.	Bhutta et al. [65]	Fibers type	Pullout test SEM	Fly ash class F	Steel (length deformed, straight, end deformed). PP(straight, length deformed).	The fracture of GP mortar reinforced with length-deformed steel fibers before being fully taken out does not represent fibers due to the brittleness of GP mortar and significant fiber deformation. -slippery-mortar-bonding-behavior.

## Data Availability

Not applicable.
